# Convergence of dopamine and glutamate signaling onto striatal ERK activation in response to drugs of abuse

**DOI:** 10.3389/fphar.2013.00172

**Published:** 2014-01-08

**Authors:** Emma Cahill, Marine Salery, Peter Vanhoutte, Jocelyne Caboche

**Affiliations:** ^1^UMRS 952, INSERM, Physiopathologie des Maladies du Système Nerveux CentralParis, France; ^2^UMR7224, CNRS, Physiopathologie des Maladies du Système Nerveux CentralParis, France; ^3^University Pierre and Marie Curie-Paris 6Paris, France

**Keywords:** receptors, addiction, dopamine, crosstalk, signaling, ERK, GPCR, striatum

## Abstract

Despite their distinct targets, all addictive drugs commonly abused by humans evoke increases in dopamine (DA) concentration within the striatum. The main DA Guanine nucleotide binding protein couple receptors (GPCRs) expressed by medium-sized spiny neurons of the striatum are the D1R and D2R, which are positively and negatively coupled to cyclic adenosine monophosphate (cAMP)/protein kinase A (PKA) signaling, respectively. These two DA GPCRs are largely segregated into distinct neuronal populations, where they are co-expressed with glutamate receptors in dendritic spines. Direct and indirect interactions between DA GPCRs and glutamate receptors are the molecular basis by which DA modulates glutamate transmission and controls striatal plasticity and behavior induced by drugs of abuse. A major downstream target of striatal D1R is the extracellular signal-regulated kinase (ERK) kinase pathway. ERK activation by drugs of abuse behaves as a key integrator of D1R and glutamate NMDAR signaling. Once activated, ERK can trigger chromatin remodeling and induce gene expression that permits long-term cellular alterations and drug-induced morphological and behavioral changes. Besides the classical cAMP/PKA pathway, downstream of D1R, recent evidence implicates a cAMP-independent crosstalk mechanism by which the D1R potentiates NMDAR-mediated calcium influx and ERK activation. The mounting evidence of reciprocal modulation of DA and glutamate receptors adds further intricacy to striatal synaptic signaling and is liable to prove relevant for addictive drug-induced signaling, plasticity, and behavior. Herein, we review the evidence that built our understanding of the consequences of this synergistic signaling for the actions of drugs of abuse.

## INTRODUCTION

Drug addiction can be considered as a chronic and relapsing psychiatric disorder induced by repeated pharmacological manipulation of the so-called mesolimbic reward circuitry by drugs of abuse. It can be viewed as maladaptive neural plasticity that occurs in vulnerable individuals in response to repeated exposure to drugs. Thus, addictive drugs change brain properties that normally permit us to adapt to environmental stimuli (Kelley, 2004; Everitt and Robbins, 2005). By changing motivational circuitry, addictive drugs progressively orient behavior toward drug-seeking and drug-taking strategies that are life-long behavioral changes (Hyman et al., 2006). The persistence of these behavioral changes relies on alterations in gene expression, an important mechanism by which chronic exposure to a drug of abuse elicits long-lasting plasticity in the brain. All drugs of abuse enhance extracellular dopamine (DA) levels in the forebrain, especially in the ventral part of the striatum, the nucleus accumbens (NAcc; Di Chiara and Imperato, 1988), where they control transcription and new protein synthesis (Robinson and Nestler, 2011) within medium-sized spiny neurons (MSNs), the major striatal neuron population. These DA inputs are normally activated in response to unpredicted rewards (Schultz, 2010), and control glutamatergic striatal inputs that are informative of the context. By hijacking DA release, drugs of abuse drive cortico-striatal plasticity (Wickens, 2009). The search for therapeutic targets to treat addiction has been recently encouraged by the discovery of new cellular and molecular substrates of DA-driven signaling in the striatum.

The main DA GPCRs expressed by MSNs of the striatum are the D1R and D2R, which are positively and negatively coupled to cyclic adenosine monophosphate (cAMP)/protein kinase A (PKA) signaling, respectively. However, besides these classical signaling pathways, it now becomes evident that direct and indirect interactions between DA-R and glutamate receptors are the molecular basis by which DA controls striatal plasticity and behavior induced by drugs of abuse. The extracellular signal-regulated kinase (ERK) signaling pathway, which is activated in a DA-D1R and NMDAR dependent manner in MSNs in response to drugs of abuse, forms the central core of striatal plasticity and adaptive behaviors in response to these drugs (Girault et al., 2007). We review herein evidence from the literature indicating that D1R-glutamate convergence onto ERK signaling in the striatum is a critical event onto striatal plasticity in response to drugs of abuse.

## STRIATAL MSN AT THE JUNCTION OF DA AND GLUTAMATE TRANSMISSION

### THE DA AND GLUTAMATE HYPOTHESES OF ADDICTION

Despite their different principal targets of action, common drugs of abuse have a shared secondary consequence on DA transmission (Di Chiara and Imperato, 1988). Dialysis for extracellular levels of DA in the striatum revealed large increases from basal levels by administration of drugs abused by humans including cocaine, amphetamine, and morphine. This increase in DA levels is significantly higher in the ventral region, NAcc, than in the dorsal part. Both regions receive dense DA afferents from the ventral tegmental area (VTA). In a physiological context, DA transmission in the striatum influences motor control, decision making, attention and working memory, and learning of reward associated stimuli (Robbins and Everitt, 2002). Therefore, it is believed to be involved in disorders where these processes are perturbed such as Parkinson’s disease (PD), Huntington’s disease, attention deficit hyperactivity disorder (ADHD), schizophrenia, Tourette’s syndrome as well as addiction. The DA hypothesis of addiction has gained significant support but the precise role of DA in reward anticipation and motivation is still under intense investigation (Wise and Bozarth, 1987). DA transmission is not believed to register the context of the drug experience, rather it is an associative learning performed by cortical and subcortical glutamatergic innervations to the striatum, which supports this (Hyman et al., 2006). While an acute treatment with drugs such as cocaine does not drastically alter the concentration of glutamate liberated in the striatum, the repeated administration or self-administration of drugs results in a potentiated glutamate availability at the synapse (Pierce et al., 1996; Zhang et al., 2001). This loss of control of glutamate transmission has been proposed to underlie the shift in behavioral control that is seen after repeated drug treatment (Kalivas, 2009). Importantly these two important transmission systems engaged by drugs of abuse, namely DA and glutamate, converge anatomically in the striatum.

### MEDIUM-SIZED SPINY NEURONS INTEGRATE DA AND GLUTAMATE SIGNALS

The DA and glutamatergic inputs converge onto the dendrites of the major cell type of the striatum, the MSN. The GABAergic MSNs are generally divided into two groups, based in part on their expression of either the DA D1 receptor (the D1R) or the D2 receptor (D2R; Gerfen et al., 1990). Each MSN population controls the striatal inhibitory output to different basal ganglia structures forming the direct, D1 expressing, and indirect, D2 expressing, striatal projection pathways and are both acted on by exposure to drugs of abuse (Smith et al., 2013). The MSN of the direct pathway inhibit the substantia nigra reticulata (SNr) neurons, which are GABAneurons with high basal firing rate. The MSNs of the indirect pathway have an opposite effect because of their inhibitory link formed by projections to the external globus pallidus (GPe; GABergic), which in turn projects to the subthalamic nucleus (STN; Glutamatergic). Therefore the indirect pathway leads to the desinhibition of SNr. Activation of the direct striatonigral pathway desinhibits thalamocortical neurons. The indirect pathway has the opposite effect. DA reinforces the direct pathway and inhibits the indirect pathway, leading to harmonious functions of the basal ganglia. Recent evidence, using genetically encoded calcium sensors, has confirmed original assumptions that a balance exists between the direct and indirect pathway and that generation of movements requires the initiation and selection of wanted motor behaviors (Carlezon et al., 1998; Cui et al., 2013). This is supported by studies where either population were artificially activated using light activated Channel rodopsin. Impressively, when the direct pathway was activated in this manner the mice produced more ambulation in an open field and, accordingly, the activation of the indirect pathway lead to an inhibition of movement (Kravitz et al., 2010). Both pathways are acted on by DA, and while a combinatorial effect is undoubtedly central for the actions of drugs of abuse many studies have highlighted the antagonist relationship between them (recently reviewed Lobo and Nestler, 2011). In general terms, the activation of the D1R expressing MSNs promotes initiation of motor behaviors along with drug reward and sensitizing properties, while the D2R seem to exert an inhibitory influence on these behaviors.

### STRIATAL DA RECEPTORS

Dopamine transmission is detected by Guanine nucleotide binding protein couple receptors (GPCRs), also known as seven-transmembrane receptors. These are metabotropic receptors share features such as the interaction with G-proteins, from which they gain their name, and the seven alpha-helices transmembrane domain structure that are interconnected by alternating intracellular and extracellular loops. The heterotrimeric G-proteins are formed by a combination of an α-subunit and βγ dimer, that can each lead to activation of signaling effectors. Exactly how the DA receptors couple to the G-proteins is not yet fully understood, but two models have been proposed (Lohse et al., 2008).

The striatum contains all the subtypes of DA GPCR to different extents (Missale et al., 1998). Since the late 1970s the DA receptors have been subdivided into two families, originally based on the prediction that one family is positively coupled to adenylyl cyclase via Gs proteins and cAMP production, and the other to its inhibition via Gi proteins (Kebabian and Calne, 1979). After the identification of D1 and D2 receptors came the molecular cloning and the subsequent discovery of a D3, D4, and D5 (previously known as the D1b in the rat). The D1R, D2R (Le Moine and Bloch, 1995), and D5R (Rivera et al., 2002) are expressed both in the dorsal and ventral striatum, while the D3 is preferentially expressed in the ventral striatum (Lévesque et al., 1992). The D4R, relatively speaking, is not as strongly expressed but is detectable in the striatum (Ariano et al., 1997).

#### Focus on striatal D1R: regulation by drugs of abuse

As already stated, there is a segregation of D1R and D2R expression in the two populations of MSNs. These two founding members of the DA receptor family are GPCR that are distinguished on the basis of their coupling to cAMP production and PKA-mediated signaling (Neve et al., 2004). Although a population of MSN exists that co-express D1R and D2R, the exact percentage seems to depend on the method of analysis, however, estimates are in the region of 17% in the ventral striatum (Bertran-Gonzalez et al., 2008). Accumulated evidence suggests that from the first drug exposure, essential signaling, and transcriptional events necessary for drug-induced alterations in behavior are set in motion primarily in D1R + MSN of the “direct” or striato-nigral, pathway (Valjent et al., 2000; Bertran-Gonzalez et al., 2008).

The D1R is expressed in the major brain regions of the reinforcement learning circuitry (i.e., the cerebral cortex, limbic system, thalamus) and within the striatum it is strongly expressed in both dorsal and ventral regions. At the cellular level, the D1R localizes principally along the perisynaptic neck regions of dendritic spines (Huang et al., 1992). A similar conclusion was drawn from immunohistochemical analysis of cortical tissue from humans and primates where the D1R signal was more “extrasynaptic” (Smiley et al., 1994). In striatal spines, the D1R come into close proximity with the glutamate receptors at the post synaptic density (PSD), and they are extensively interconnected via scaffold proteins and intracellular signaling proteins.

Pre-treatment with SCH23390, a D1R antagonist, inhibited acute cocaine-induced locomotor activity and prevented the development of locomotor sensitization in a “one-shot” protocol (Fontana et al., 1993; White et al., 1998). Later studies confirmed that SCH23390 but not D2R antagonist raclopride, prevented the development of locomotor sensitization to cocaine, and furthermore a contribution of the NMDAR to this behavior was identified (Valjent et al., 2000).

Two lines of D1R KO mice lacked the acute locomotor and stereotyped behaviors normally induced by cocaine (Xu et al., 1994; Miner et al., 1995). This altered response was specific to D1R homozygous knockout mice since heterozygous mice still responded (Miner et al., 1995). In a further study, the chronic effects of cocaine were also perturbed, as the animals did not show sensitization of the locomotor activity after repeated cocaine administration (Xu et al., 2000). However, the D1R KO have hyper basal-locomotor activity, which remained elevated in these studies, so the use of a locomotor readout for cocaine’s effects may not be as relevant as other measures. Although D1R KO mice developed a normal conditioned place preference (CPP) to cocaine (Miner et al., 1995), they did not develop self-administration of cocaine, despite having learned sufficiently the operant association for food (Caine et al., 2007). The D1R contribution to self-administration therefore appeared to be function of the reward and whether context or operant reward learning is recruited relied differentially on functional D1R.

### STRIATAL GLUTAMATE RECEPTORS

The striatum receives vast glutamatergic inputs from cortical and subcortical regions, which converge anatomically with the DA inputs. Early studies used various antagonists of glutamate receptors to show role for glutamate in the development of drug-induced behaviors (Karler et al., 1989), and lesion studies confirmed this contribution came from the glutamate circuitry coming from hippocampus, the PFC, and amygdala onto the NAc (for review, Wolf, 1998). In MSNs, ionotropic and metabotrophic glutamate receptors are expressed in close proximity to the DA GPCR (Hara and Pickel, 2005). This morphological co-localization of DA and glutamate receptors provides a number of possibilities for interactions, including signaling crosstalk and direct interaction in protein complexes.

#### Metabotropic glutamate receptors

Glutamate, like DA, can also activate GPCRs. Eight mGluRs are classified into three groups based on sequence homology and G protein interactions: group I (mGluR1, mGluR5), group II (mGluR2, mGluR3), and group III (mGluR4, mGluR6, mGluR7, and mGluR8; Ferraguti and Shigemoto, 2006).

mGluR1 and mGluR5, which are Gq protein-coupled, are mostly located postsynaptically (Mitrano and Smith, 2007). Their activation induces mobilization of intracellular Ca^2^^+^ stores and activation of phospholipase C (Schoepp and Conn, 1993). mGluR5 KO animals do not self-administer cocaine and do not display locomotor sensitization (Chiamulera et al., 2001). Systemic administration of the mGluR5 receptor antagonist, MPEP, decreased cocaine self-administration (Kenny et al., 2003, 2005; Lee et al., 2005; Platt et al., 2008) and attenuated the ability of a priming injection of cocaine (Lee et al., 2005) or cocaine-associated cues (Bäckström and Hyytiä, 2007) to reinstate cocaine seeking. Administration of MPEP into the NAcc shell attenuated cocaine priming-induced reinstatement of drug seeking, an animal model of relapse (Kumaresan et al., 2009). Altogether these data clearly indicated that activation of mGluR5, specifically in the NAcc, may promote the reinstatement of drug seeking.

The activation of the presynaptic Group II mGluRs inhibits cAMP and PKA signaling as they are coupled to Gi/o proteins. The reduction in extrasynaptic glutamate availability after chronic cocaine treatment and withdrawal, due to disruption of the cysteine-glutamate exchanger, removes the tonic activation of group 2 mGluR that would normally inhibit glutamate release. This can explain why during reinstatement, the challenge, or renewed drug experience or cue evokes a potentiated glutamate response, which is not seen with an acute injection of cocaine (Pierce et al., 1996).

The group 3 mGluR contain mGluR4, mGluR6, mGluR7, and mGluR8 and similarly to Group 2 they are negatively coupled to adenyl cyclase activity and found presynaptically in the glutamatergic terminals of the striatum. When an agonist (L-AP4) of the group 3 mGluR was administered into the striatum of cocaine-naïve mice there was no effect on locomotor activity, however, in mice subsequently treated with cocaine the compound was able to prevent the induction of locomotor sensitization (Mao and Wang, 2000).

#### Focus on NMDAR: regulation by drugs of abuse

The ionotropic glutamate receptors are classed based on their affinity for synthetic agonists: *N*-methyl-D-aspartate (NMDA), α-amino-3-hydroxy-5-methy-4-isoxazole propionate (AMPA), and kainate. These are ligand gated ion channels, composed of heteromeric complexes of four integral membrane protein subunits and have long intracellular cytoplasmic tails. Unlike the AMPAR or kainate receptors, the NMDAR channel is blocked by the presence of a magnesium ion in a voltage-dependent manner. In this way they respond to the binding of glutamate only when accompanied by a depolarization of the post synaptic membrane (Mayer et al., 1984). Furthermore their activation requires the binding of a co-agonist (Kleckner and Dingledine, 1988). The NMDAR are permeable to monovalent cations and calcium, and so provide a major entry point for triggering Ca^2^^+^dependent intracellular pathways. The NMDAR subunits are grouped into classes: GluN1, GluN2A-D, and GluN3A-B (formally denoted NR1, etc.). The different subunits contain different agonist binding sites, which infer them each with a specific pharmacology. The GluN1 or GluN3 are obligatory to form the ion channel and contain the binding site for the co-agonists glycine (Kleckner and Dingledine, 1988) or D-serine (Mothet et al., 2000) and it is the GluN2 subunits that contain the site for glutamate (Laube et al., 1997).

Context-dependent sensitization induced by single exposure to cocaine is completely prevented in mice pre-treated with the selective NMDAR antagonist, MK801, in a two-injection protocol (Valjent et al., 2010), as well as in the repeated injection protocol (Schenk et al., 1993). Similarly, GluN1-knockdown mice showed an attenuation of sensitization induced by cocaine (Ramsey et al., 2008). The precise location at which NMDA receptors are critical appears not to be limited to DA neurons themselves. Mice with specific inactivation of GluN1 in DA neurons did not show alteration of short-term sensitization, but a decreased long-term sensitization (Engblom et al., 2008; Zweifel et al., 2008). In contrast, expression of mutant NMDARs in D1R-containing MSNs prevented cocaine sensitization (Heusner and Palmiter, 2005). Altogether, these observations support the hypothesis that NMDARs located in MSNs, in the striatum, and/or on their terminals in the VTA, as indicated by the effects of local infusion of antagonists (Vezina and Queen, 2000), contribute to the development of sensitization.

One day after acute cocaine treatment the total expression levels of the NMDAR subunits do not change in the NAcc, however, their subcellular localization is altered, with an increased internalization (Schumann and Yaka, 2009). After a 3-weeks withdrawal from repeated cocaine exposure, global expression levels and surface expression of the NMDAR subunits are increased, specifically in the NAcc. This indicates that during withdrawal some long-term processes are occurring to heighten the glutamatergic receptor levels in the NAcc.

## SIGNALING CROSSTALK BETWEEN D1R AND GLUTAMATE RECEPTORS

### D1R-MEDIATED PKA REGULATION AND ITS INFLUENCE ON GLUTAMATE RECEPTORS

An acute injection of cocaine does not increase the levels of D1R protein in the dorsal striatum but trigger intracellular signaling cascades that are capable of modulating cell excitability via interactions with ion channels, including the glutamatergic AMPAR and NMDAR. In the striatum, as the D1-like family of DA receptors are coupled to stimulatory G_α*solf*_ G-proteins (see Hervé, 2011, for review), the most widely studied consequence of G-protein activation downstream of DA receptors is their influence on PKA-regulated signaling.

After the binding of DA to D1R a change in G-protein association enables the activation of the Ca^2^^+^-insensitive adenylyl cyclase 5 (AC5) isoform. The major target of cAMP is the cAMP-dependent PKA that has many targets including the glutamate receptor subunits. The duration of PKA activation is determined by feedback loops due to the activation of phosphodiesterases (PDEs) that are expressed in the striatum and limit cAMP production (Menniti et al., 2006).

Protein kinase A rapidly phosphorylates the NMDAR (Leonard and Hell, 1997) in response to DA, even after just 30 s in presence of a DAT inhibitor (Snyder et al., 1998). This phosphorylation occurs at Ser^897^of the GluN1 subunit (Tingley et al., 1997). Phosphorylation of NMDAR subunits is a well-characterized mechanism to control their trafficking to the membrane (Scott et al., 2001; Lau and Zukin, 2007). Ion channels are also targeted by PKA and their phosphorylation can alter the conductance state of the cell. PKA mediated phosphorylation of sodium channels leads to hyperpolarization of MSNs (Schiffmann et al., 1995), and can indirectly diminish N and P/Q-type channel calcium currents that are largely localized to dendrites. On the other hand, L-type currents, at the soma, are potentiated by a PKA mechanism that boosts cellular conductance (Surmeier et al., 1995). Antagonists of the L-type Ca^2^^+^ channels can prevent the reinstatement of cocaine seeking, this was linked to the activation of Ca^2^^+^/CaM-dependent kinase CaMKII and regulation of AMPAR trafficking (Anderson et al., 2008).

### PKA REGULATION OF DARPP-32 AND INFLUENCE ON GLUTAMATE RECEPTORS

A number of the intermediates between PKA and its transmembrane protein targets are kinases and/or phosphatases particularly enriched in the striatum. In the late 80s, the Greengard group characterized many of these, including ARPP-16 (cAMP-regulated phoshphoprotein of Mr 16), ARPP-19, ARPP-21 (regulator of calmodulin signaling), ARPP-39, and ARPP-90 (Rap1Gap, Walaas et al., 1989; Girault et al., 1990; Walaas and Greengard, 1993). ARPP-16 and ARPP-19 phosphorylation was confirmed to be strictly dependent on the D1R (Dulubova et al., 2001). DA and cAMP regulated phosphoprotein of Mr 32kDa (DARPP-32) was identified before many of the ARPPs and has received more attention in the context of drugs of abuse as the expression of DARPP-32 is most evident in neurons of the ventral and dorsal striatum (Walaas et al., 1983).

In the context of addiction, the knockout mice for DARPP-32 had diminished hyper-locomotor responses at low doses of acute cocaine (Fienberg et al., 1998; Hiroi et al., 1999). Locomotor sensitization to cocaine was absent in the DARPP-32 knockout when a two injection protocol of sensitization (TIPS) was used (Valjent et al., 2005) but not after repeated injections (Hiroi et al., 1999). PKA phosphorylates the Thr^34^ residue of DARPP-32 that permits it to act as an inhibitor of protein phosphatase I (PP1, Hemmings et al., 1984). By this means, DARPP-32 supports PKA driven activity, in particular certain substrates such as the phosphorylation of GluR1 at Ser^845^ and GluN1 at Ser^897^. Additional phosphorylation sites regulate DARPP-32 activity including Thr^75^ by Cdk5, Ser^97^ by CK2, and Ser^130^by CK1 (see Walaas et al., 2011, for review). The Thr^75^ phosphorylation allows DARPP-32 to regulate its own activity by inhibiting PKA and, in basal conditions this site is phosphorylated. The Ser^97^ site is located near a nuclear export signal and aids DARPP-32 to remain outside the nucleus when phosphorylated (Stipanovich et al., 2008). The Ser^130^residue also allows DARPP-32 to regulate its own activity because it inhibits the dephosphorylation of the Thr^75^ site by protein phosphatase 2B (PP2B). The gene encoding DARPP-32, *ppp1r1b*, is subject to polymorphisms in humans that gives rise to a truncated form (t-DARPP-32), which lacks the Thr^75^ site and is associated with schizophrenic and bipolar phenotypes (Kunii et al., 2013). These sites are equally subject to dephosphorylation. Thr^34^ is dephosphorylated by the CaN and thereby allows the activity of PP1 (Nishi et al., 1999). On the other hand, dephosphorylation of Thr^75^ by PP2A, favors the phosphorylation of Thr^34^ and PP1 inhibition. PP2A also dephosphorylates the Ser^97^ residue. In the striatum, PP2A is activated after cAMP-induced phosphorylation of its B56δ subunit.

One extensive study systematically assessed the role of the different residues in DARPP-32 by performing point mutations that replaced the sites that would be normally be phosphorylated by an alanine (Stipanovich et al., 2008). A notable effect was that the Ser^97^ site in itself controls the subcellular localization of DARPP-32. When Ser^97^was mutated to alanine, DARPP-32 was unable to be exported from the nucleus to the cytoplasm and the opposite was observed if a phosphorylation-mimicking mutation, to aspartate, was made. Both these effects were preserved even after activation of the D1R by SKF81297. To investigate the Ser^97^ mutation to alanine *in vivo*, mutant mice were also generated and analyzed for alterations in cocaine-induced behaviors (Stipanovich et al., 2008). Firstly, the Ser^97^Ala-DARPP32 mice had normal acute locomotor responses to cocaine but a reduced locomotor sensitization to a second cocaine injection. Secondly, cocaine-induced CPP was not observed in these mice. Finally, this mouse line had a lower breaking point in a food self-administration test. These findings suggested that the nuclear shuttling of DARPP-32 is particularly important for the learned associative behaviors for reward seeking. The contribution of DARPP-32 in locomotor behaviors was further investigated in conditional knockout mice for DARPP-32 expression in either the D1R-expressing or D2R-expressing MSNs (Bateup et al., 2010). The loss of DARPP-32 in either population prevented the induction of long term potentiation (LTP). The conditional knockout in D1R MSNs had a diminished basal locomotor activity as measured by the distance traveled in an open field maze, while a disinhibition of activity was seen in the D2R conditional knockout. The lack of effect on basal locomotion in the full knockout may be interpreted as the net result of this D1R-MSN hypo-locomotor activity plus the D2R-MSN driven hyperactivity.

### D1R MODULATION OF NMDAR SIGNALING VIA SFK

When Wittman and colleagues investigated the potentiation of NMDAR responses by clozapine (a last-resort medication for schizophrenia which has multiple actions including on the DA and Glu limbic system), they found that PKA inhibitors prevented the D1R enhancement of NMDAR transmission. Similar results were also found in presence of “PP2,” an inhibitor of the Src family kinases (SFKs). They went on to show that the D1R stimulation potentiated calcium influx via NMDAR containing the GluN2B subunit (Wittmann et al., 2005). The SFK members expressed in the CNS are Src itself, Fyn, Lyn, Lck, and Yes. Src and Fyn are the most homologous in sequence and are both found in the PSD, where they potentiate NMDAR currents (Köhr and Seeburg, 1996).

Using subcellular fractionation, Dunah and Standaert (2001) observed that the GluN2B and GluN2A contents of synaptosomal striatal membranes were increased after stimulation of the D1R and this was dependent on the action of protein tyrosine kinases. GluN2B can be phosphorylated by SFK/Fyn *in vivo* (Nakazawa et al., 2001), and D1R-induced enhancement in the synaptic content of NMDAR subunits is absent in Fyn knockout mice (Dunah et al., 2004). Yaka et al. (2002) noticed sequence homology between certain regions in Fyn and GluN2B-ct and described a binding interaction between these two sites with the scaffold protein RACK1 that limits the ability of Fyn to phosphorylate GluN2B. In a follow up study, the authors further characterized the implicated pathways and found that, in the hippocampus, activation of the pituitary adenylate cyclase activating peptide (PACAP) receptors stimulated Gs signaling that releases RACK1 from GluN2B and Fyn and increased NMDAR activity. This was associated with PACAP-induced translocation of RACK1 to the nucleus where it induced brain derived neurotrophic factor (BDNF) expression (Yaka et al., 2003). An interaction also exists between Fyn and PSD-95, since the Src homology 2 (SH2) domain of Fyn binds to the third PDZ domain of PSD-95 (Tezuka et al., 1999). PSD-95 associates with the last four amino acids of the GluN2B subunit, which are in close proximity to the Tyr^1472^ residues targeted by Fyn. In this way, PSD-95 may bring Fyn nearer to the GluN2B subunit for its phosphorylation and also regulate GluN2B trafficking. D1R stimulation results in an increase in Fyn colocalization with GluN2B, which is Fyn (and not Src) dependent in the PFC (Hu et al., 2010).

Importantly, it was recently shown that D1R and NMDAR co-stimulation, as well as cocaine *in vivo*, activated Fyn and phosphorylated GluN2B at the Tyr^1472^ residue. The activation of Fyn was G-protein dependent, but independent of cAMP production (Pascoli et al., 2011a). The exact mechanisms governing the activation of Fyn in the striatum are, however, not fully understood. The SFK are found in an inactive state when they are phosphorylated on Tyr^525^ (for mouse Fyn). The dephosphorylation of this site and autophosphorylation at Tyr^420^ leads to a conformational change that opens the activation loop. The inactive conformation involves a binding interaction between the Fyn SH2 and SH3 domains (for review see Salter and Kalia, 2004). Therefore, the above-mentioned interaction of PSD-95 with the SH2 domain of Fyn may favor Fyn activation, but whether it may be a cause or consequence is unclear.

As for the deactivation of Fyn an interesting candidate is striatal enriched protein tyrosine phosphatase (STEP). STEP dephosphorylates GluN2B at Tyr^1472^(Snyder et al., 2005). STEP exists in two major catalytic isoforms STEP46 and STEP61, which have distinct N-terminus regions, and each isoform has a specific intracellular location. STEP46 is localized in the cytosolic compartment, while STEP63 is attached to membranes of the ER or PSD. To interact with its substrates, STEPs must bind them using a kinase interacting motif (KIM) domain. The KIM of STEP is phosphorylated by PKA after D1R activation, at Ser160 and Ser221 in STEP61 and Ser49 for STEP46 (Paul et al., 2000). This phosphorylation event prevents STEP binding to its substrates. The Ser160 site is suspected to have a role in the regulation of a proteolytic cleavage sequence (known as PEST sequences) in STEP, whereas Ser221 and Ser46 may lose affinity for their substrates after phosphorylation. STEP61 KIM binds to the SH2 and N-terminus domain of Fyn, and dephosphorylates the Tyr^420^ regulatory site but not the Tyr^531^ sites (Nguyen et al., 2002). STEP also dephosphorylates the mitogen activated protein kinase (MAPK) extracellular-signal regulated kinase (ERK; Pulido et al., 1998).

### EXTRACELLULAR SIGNAL-REGULATED KINASE PATHWAY

A major consequence of drugs of abuse administration is activation of the MAP kinase/ERK (extracellular-signal regulated kinase) pathway. ERK1 and ERK2 are two closely related mitogen-activated protein kinases (MAP-kinases), which are activated by phosphorylation of their activation loop by MAP-kinase and ERK-kinase (MEK 1 and 2). Valjent et al. (2000) demonstrated that acute cocaine elicits a rapid and transient increase in ERK1/2 phosphorylation within the ventral and dorsal striatum that remained after chronic administration. They further demonstrated that ERK1/2 activation was a common feature of most drugs of abuse (Valjent et al., 2001, 2004). Even if slight differences could be observed in the kinetic patterns of activation, nicotine, morphine, Δ9-tetrahydrocannabinol (THC) and psychostimulants, increased ERK1/2 phosphorylation occurred in the reward circuitry, including the prefrontal cortex, the striatum (dorsal part and NAc) as well as the extended amygdala. In all cases, and all structures drug-induced ERK activation was blocked by the D1R antagonist, SCH23390 (Valjent et al., 2004). 3,4-methylenedioxy-methamphetamine (MDMA, also known as ecstasy), and ethanol also activate ERK1/2 in the striatum (Salzmann et al., 2003; Ibba et al., 2009). ERK1/2 activation induced by drugs of abuse is functionally relevant since SL327, a pharmacological compound that act on MEK and crosses the blood-brain barrier, prevented the long-term behavioral effects of cocaine, such as CPP; Valjent et al., 2000, or the development of locomotor sensitization (Valjent et al., 2006a). A different MEK inhibitor, PD98059, injected into the NAcc either before or after CPP training sessions blocks subsequent amphetamine CPP expression (Gerdjikov et al., 2004). ERK activation is also involved in the reconsolidation of drug-associated memories since it was reactivated by exposure to the drug-associated context and because MEK inhibition was able to erase previously acquired CPP (Miller and Marshall, 2005; Valjent et al., 2006b).

Striatal ERK2 activation involves both D1Rs and NMDA glutamate receptors, since it is prevented by either a D1R antagonist or in D1R knock-out mice, or by an NMDA antagonist (Valjent et al., 2000, 2005). It is only observed in a subset of D1R-expressing striatonigral neurons (Bertran-Gonzalez et al., 2008). Therefore, the modality of ERK1/2 activation in MSNs has been proposed to reflect the convergence of DA and glutamate signaling onto MSNs, thereby placing ERK1/2 as a coincidence detector, which detects the simultaneous arrival of contextual information coded by corticostriatal and thalamostriatal glutamate inputs, and the reward prediction error coded by DA neurons (Girault et al., 2007).

Whether ERK1 or ERK2 is fully responsible for the molecular responses to cocaine remains to be established. Ablation of ERK1 in cultured cells resulted in a stimulus-dependent increase of ERK2 signaling (Mazzucchelli et al., 2002), without altering the basal levels of ERK2 expression. This apparent competition of ERK1 with ERK2 signaling, that is removed in the knock-out mice, lends the interpretation of the independent role of each isoform complex. However, the generation of ERK1 mutant mice revealed that removal of ERK1 results in an hypersensitivity to the rewarding properties of morphine and the rewarding and psychomotor effects of cocaine (Mazzucchelli et al., 2002; Ferguson et al., 2006). Furthermore, increased synaptic plasticity LTP was observed in the NAcc, hippocampus, and lateral amygdala slices from ERK1 KO mice, an effect that was specifically reversed by U0126, a selective MEK inhibitor (Mazzucchelli et al., 2002). These findings strongly support that ERK2 is the dominant isoform for neuronal plasticity and behavioral adaptations induced by addictive drugs.

**FIGURE 1:**
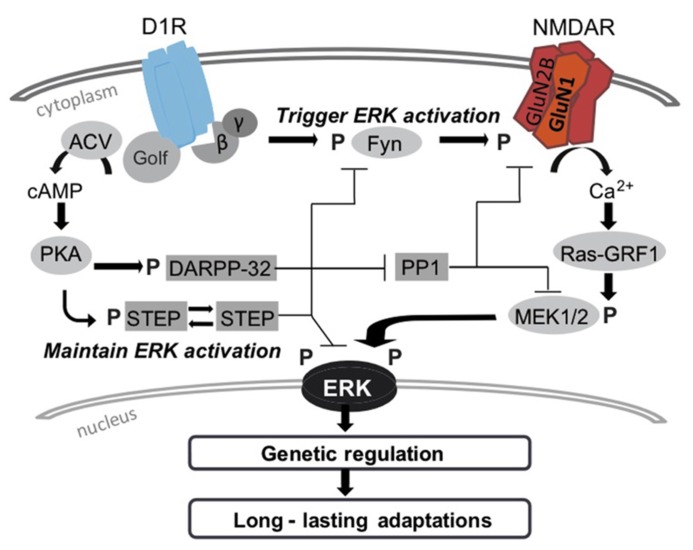
**Cocaine, similarly to most drugs of abuse, produces long lasting brain changes, that relie on gene expression and new protein synthesis.** These long-term neuronal adaptations take place in majority in DA-D1R expressing MSNs that belong to the direct pathway. The ERK pathway is central in this striatal plasticity, and detects a combination of glutamate and DA signals that are essential for long lasting modifications. Cocaine-induced ERK activation depends on complex cascade of phosphorylation events downstream D1-R. Importantly, the triggering event in ERK activation depends on a non-canonical signaling pathway that associates Fyn-induced phosphorylation of GluN2B and increases in calcium influx into D1-R MSNs. The duration and maintenance of ERK activation occurs via cAMP/PKA pathway. The cAMP/PKA pathway, downstream D1 receptors (left panel) triggers deactivation of phosphatases, PP1 on one hand via DARPP-32, and STEP on the other hand (Valjent et al., 2005). By controling the activity of the tyrosine kinase Fyn and the dual specificity protein kinase MEK, this signaling cascade intervenes in the state of phosphorylation of ERK downstream DA-D1R stimulation. However, we propose that triggering ERK activation depends on a “non-canonical signaling” pathway downstream D1-R (right panel). A cAMP-independent activation of Fyn produces tyrosine phosphorylation of GluB2B subunit of NMDA-Rs that in turn facilitates calcium influx (Pascoli et al., 2011a), and activation of the calcium-sensitive Ras-guanine releasing factor (Ras-GRF1) that activates the MEK/ERK pathway (Fasano and Brambilla, 2011). Upon cocaine stimulation ERK translocates to the nucleus where it controls epigenetic and genetic programs. Among the latter, the transcription factor Elk-1 is a component of the ternary complex factor, which binds to SRE (Besnard et al., 2011a). ERK1/2 also phosphorylates the nuclear kinase MSK1which appears to play a prominent role in phosphorylation of histone H3 and cAMP-response element-binding (CREB) protein (Brami-Cherrier et al., 2005). This leads to the expression of immediate-early genes, which are particularly sensitive to CREB (e.g., cFos) or ternary complex factor (e.g., Zif268 a.k.a. Egr-1). These two nuclear pathways downstream from ERK have distinct roles in long-term behavioral adaptations (see text). DA-GPCR and NMDAR, in particular D1R and GluN1, oligomerization has been documented in heterologous systems, hippocampal, and striatal tissues after cocaine exposure. The role of D1R-GluN1 interaction in ERK activation and downstream molecular events remains to be established.

Upstream from MEK, ERK activation induced by cocaine involves the calcium-activated guanine nucleotide exchange factor Ras-GRF1 (Fasano et al., 2009; Fasano and Brambilla, 2011). Knock-out mice for Ras-GRF1 show a significant reduction, but not total inhibition, of cocaine-induced ERK activation, and locomotor sensitization. On the other hand, mice that overexpressed Ras-GRF1 were more sensitive to cocaine treatment than wildtype counterparts (Fasano et al., 2009; Fasano and Brambilla, 2011; Cerovic et al., 2013). Interestingly, Ras-GRF1 is preferentially associated with GluN2B subunits (Krapivinsky et al., 2003). The cross talk between D1R and NMDA receptors implicates a cAMP-independent pathway that increases responsiveness of GluN2B containing NMDA receptors to glutamate (Pascoli et al., 2011a). In striatal neurons, D1R stimulation leads to an increase in Ca^2^^+^ influx through NMDARs via SFK/Fyn-induced phosphorylation of the GluN2B subunit at Tyr^1472^. In parallel, PKA-mediated phosphorylation of DARPP-32 promotes ERK activation through an indirect inhibition of STEP. DARPP-32 thus prevents ERK dephosphorylation and contributes to enhanced glutamate mediated-ERK activation (see **Figure 1**; Valjent et al., 2005).

As stated above, mGluR5 receptors seem to be critically involved in cocaine-induced long term behavioral alterations. A link between amphetamine-induced ERK phosphorylation and mGluR5 was found when using a mGluR5 antagonist, MPEP, which blocked amphetamine induction of ERK in the dorsal striatum (Choe et al., 2002). Surprisingly, this inhibition was not due to mobilization of intracellular Ca^2^^+^ stores since dantrolene – a compound that blocks intracellular Ca^2^^+^ release from ryanodine-sensitive stores – did not affect amphetamine effects. The Group I mGluR can modulate NMDAR signaling onto ERK activation via PKC (Niswender and Conn, 2010) and also via direct physical interactions (Perroy et al., 2008).

An elegant work from Pascoli et al. (2011b) recently showed that ERK activation was necessary for corticostriatal LTP induced by electrical stimulation in slices of mice treated with cocaine administration *in vivo*, and that this synaptic plasticity was necessary for locomotor sensitization (Pascoli et al., 2011b). Furthermore, it is now well established that this long-term behavioral adaptation to cocaine relies on gene regulation and new protein synthesis (Valjent et al., 2006a).

Under basal conditions, the unphosphorylated form of ERK1/2 is detected in the cytoplasm of neurons. Upon cocaine administration, the active di-phosphorylated ERKs translocate to the nucleus where they control both epigenetic and genetic responses. This cyto-nuclear shuttling of ERK involves clathrin-dependent endocytosis originating from glutamate AMPA receptors (AMPAR) endocytosis (Trifilieff et al., 2009). Within the nucleus ERK2 can phosphorylate several nuclear kinases, including p90 ribosomal protein S6 kinases (RSK1/2) and the mitogen and stress-activated protein kinases MSK1 and 2. MSK1, but not RSK1/2 nor MSK2, is rapidly phosphorylated downstream of ERK in MSN in response to cocaine (Brami-Cherrier et al., 2005). In turn, activated MSK1 targets both the cAMP-responsive element-binding (CREB) transcription factor and histone H3 and *msk1* knockout mice show a decreased phosphorylation of Ser^133^-CREB and Ser^10^-histone H3 induced by cocaine (Brami-Cherrier et al., 2005). This effect on Ser^133^-CREB phosphorylation was particularly remarkable as this residue is a common substrate for various kinases, including RSK1/2, CaMKIV, and PKA, with PKA being traditionally considered as the major CREB kinase in the striatum (Nestler, 2004). For transcription to occur, DNA decompaction is required to allow the action of the core transcription machinery and transcription factors. Such remodeling of DNA depends on post-translational modifications of histones, including Ser^10^-histone H3 phosphorylation that positively regulates gene expression (Brami-Cherrier et al., 2009). The default of cocaine-induced Ser^10^-histone H3 phosphorylation observed in *msk1* deficient mice was associated with a striking impact on the induction of immediate early genes, such as c-Fos and dynorphin, which were blocked, whereas the induction of Egr-1/Zif268 was spared (Brami-Cherrier et al., 2005). Remarkably, *msk1* null mice displayed a decreased psychomotor sensitization to cocaine, whereas CPP was slightly enhanced, in agreement with the role of CREB and dynorphin in the aversive effects of drugs (Carlezon et al., 1998). MSK1-independent genes, such as *zif268*, thus seem to be more related to the rewarding properties of cocaine, as supported by the lack of cocaine-induced CPP encountered in *zif268* knockout mice (Valjent et al., 2006b). Although the role of kinases in histone H3 phosphorylation has been extensively investigated, phosphatases are also implicated in this process. Inhibition of PP1, the phosphatase for Ser^10^-histone H3, is induced by cocaine via phosphorylation and nuclear translocation of DARPP-32 and is involved in H3 phosphorylation in response to cocaine (Stipanovich et al., 2008).

Ternary complex factors (TCFs) are direct ERK targets that control serum responsive element (SRE)-driven transcription. Among TCFs, Elk1 is phosphorylated by ERK in MSN in response to acute cocaine or amphetamine (Valjent et al., 2000; Choe et al., 2002). The role of Elk1 in mediating cocaine responses was difficult to determine because of the redundancy within TCFs (Cesari et al., 2004) and because Elk-1 is found in dendrites and nuclei of neurons (Sgambato et al., 1998) where it governs opposite biological outcomes (Besnard et al., 2011b). In MSN, phosphorylation on Ser^383/389^-Elk-1 by ERK triggers its translocation from the cytoplasm to the nucleus (Lavaur et al., 2007). The selective inhibition of Elk-1 phosphorylation, downstream ERK, using a cell penetrating peptide, the TAT-DEF-Elk-1 (TDE; Lavaur et al., 2007), significantly impaired cocaine-induced IEG expression (cFos, Zif268, and Arc) along with dentritic spine formation onto MSN (Besnard et al., 2011a). An unexpected effect of TDE was found on chromatin remodeling since histone H3 phosphorylation induced by cocaine was strongly impaired, despite maintained MSK-1 phosphorylation. These data indicate that Elk-1 phosphorylation in the nucleus orchestrates important molecular events, including the recruitment of MSK-1 to the DNA, close to histones. Importantly, TDE also interfered with locomotor sensitization and CPP (in a one but not three-pairing protocol).

## OLIGOMER FORMATION: A POTENTIAL MECHANISM FOR D1R-MEDIATED REGULATION OF GLUTAMATE TRANSMISSION

Interactions between DA-GPCR and NMDAR have been documented The subcellular co-localization of D1R and NMDAR has been characterized in the striatum by electron microscopy (Hara and Pickel, 2005). This physical proximity supported the idea that the two receptors interact extensively. Lee et al. (2002) demonstrated that D1R interact with the GluN1 and GluN2A subunits, using co-immunoprecipitation studies from rat hippocampal cultures and transfection of Cos-7 cell and HEK2397 lines (Pei et al., 2004). Three GST-peptides were constructed based on the sequence of the D1R c-terminal tail (D1-t sequences) and interactions were revealed between the D1-t2 sequence and the GluN1 subunit and D1-t3 sequence and the GluN2A subunit. Subsequent biochemical studies using synthesized peptides were able to determine, by mass spectrometry, that the interaction was likely due to electrostatic forces (Woods et al., 2005). The GluN1-C1 cassette contains the Arg-rich epitope and the D1R contains a corresponding acidic epitope. Mass spectrometry showed that these two regions interacted and interestingly, they found that Ser397 in the Dt2 acidic epitope could possibly be phosphorylated by CKI and a phosphorylated version of the peptide bound five times more to the GluN1 Arg rich epitope. It remains to be shown in more native receptor environments if indeed this residue favors the association. The stability of the D1-t2/GluN1 interaction after exposure to ligand remains unclear. In hippocampal neurons and in Cos-7 cells co-transfected with D1R and GluN1, Lee et al. (2002) saw a significant decrease in the co-immunoprecipitation of D1R or Glu1 after treatment with SKF-81297. However, no such changes were seen for the D1/GluN2A interaction.

The D1R/GluN1 interaction was also found in PSD enriched fractions of striatal tissue (Fiorentini et al., 2003). The authors showed that co-expression of the D1R/GluN1/GluN2B subunits allowed their trafficking to the membrane. It had previously been established that GluN1 alone is restricted to the ER and when GluN1 was co-transfected with D1R, the D1R signal also became restraint to the cytosol and ER. It is thought that oligomerization may hide ER retention signals allowing the passage of the receptor to the export machinery. Despite DA not affecting the BRET signal for association, it was shown that SKF-81297 treatment prevented the internalization of D1R but only when co-expressed with GluN1/GluN2B. This may suggest that once at the membrane in a formed complex, stimulation of the D1R stabilized its presence or association. The association of D1R with NMDAR not only alters their trafficking from the ER to the plasma membrane but also along its surface. Scott and colleagues were able to observe that after just 3 mins from exposure to NMDA, MSN spines contained more D1R (Scott et al., 2006). To explain how D1R signal in spines was increased by NMDA, the authors proposed that the direct interaction with NMDAR would “trap” the D1R to the synapse. They performed site-directed mutagenesis experiments and found that if Ser^397^ or Ser^398^ in the Dt2 domain were mutated to alanine the NMDA effect was lost and the D1R no longer bound GluN1 GST-peptides. The authors were unable to confirm a kinase for these sites, yet their findings established a model, the “diffusion trap,” whereby NMDAR activation would cause some change in the receptor to enhance the binding with D1R and “trap” them to the spine. The ultimate result of the above mentioned diffusion trap would be to potentiate mutually the receptors.

The D1R/GluN1 oligomer has been demonstrated to have consequences on cellular plasticity and behavior in the hippocampus (Nai et al., 2010). The authors described that the D1R mediated enhancement of plasticity was via direct interactions with GluN1 that was also necessary for spatial working memory. Studies have also addressed whether these D1R/NMDAR oligomers may be implicated in pathologies. DA and glutamate transmission are frequently considered in studies of PD, and Fiorentini et al. (2006) found a significant decrease in the D1R/NMDAR interaction in both the 6-OHDA lesioned mice and in the L-DOPA induced dyskinetic (LID) mice relative to controls.

As with regards to drugs of abuse, one study did examine the D1R/GluN1 interaction after exposure to cocaine (Sun et al., 2009). By Western-blotting, the authors did not detect any change in total expression of the D1R or GluN1 in the dorsal striatum over a 60-min period following an acute (30 mg/kg) injection of cocaine. The authors reported that at 30 min post-acute cocaine there was a significant decrease in the GluN1 immunoprecipitated by the D1R antibody. The authors speculated that as the time point where they saw their effect corresponds to the point at which ERK activity returns to basal activity, the two may be linked and that the disruption of the D1R/GluR1 interaction may limit ERK activity. These possibilities were not tested and so remain to be investigated. A D2R/GluN2B oligomer was also found to be regulated by cocaine (Liu et al., 2006). In the striatum the association between the third intracellular loop of the D2R and the carboxyl terminal tail of GluN2B was enhanced by cocaine. The binding of D2R to the GluN2B displaces CaMKII and reduced GluN2B phosphorylation at Ser^1303^and calcium currents. Furthermore this interaction was shown to have functional consequences as a TAT-coupled peptide that mimicked the D2R region of interaction prevented acute horizontal activity and stereotyped behavior normally induced by cocaine. These studies suggest that direct interactions between DA GPCR and NMDAR will provide another level for interaction, and perhaps possible interventions, with regards to addictive-drug induced signaling.

## CONCLUSION PERSPECTIVES

Despite the usefulness of animal models for studies of addiction treatment the majority of therapeutic strategies for cocaine addiction still consist mostly of psychiatric therapy. For psychostimulants in general, there is a marked absence of effective treatments despite considerable neurobiological knowledge. Unlike some other “addictions” substitution regimes do not exist for cocaine. Most strategies are based on targeting the neurotransmitter receptors implicated in the reward circuitry. Dissimilar to alcohol or opiates, the withdrawal syndrome from cocaine is not as severe and thus, therapies can focus more on preventing relapse and craving rather than treating withdrawal symptoms.

As DA plays a critical role in motivation, reward, and locomotion, studies have focused on modifying its functions in addicted humans. To counter the DA increase elicited by cocaine, a DA depletor reserpine was tested in humans but found to be ineffective (Winhusen et al., 2007). A D1R antagonist Ecopipam was tested in humans who were addicted to crack cocaine, and was found to reduce self-assessed measures of acute cocaine effects such as the “high” (Romach et al., 1999). However, with chronic administration Ecopipam unfortunately failed as it actually increased the reported “high” of the drug and increased self-administration (Haney et al., 2001). An explication for these findings is that the levels of expression and D1R sensitivity may be upregulated after chronic antagonism. Targeting solely DA transmission therefore has many caveats, in addition agonists or indeed antagonists may have non-specific effects on other DA regulated functions such as body temperature or cardiovascular function and the antagonists may alter mood and lead to non-compliance.

The glutamatergic system is also implicated in cocaine addiction and therapeutic studies have turned to drugs, which should balance glutamate transmission. Unfortunately few studies have shown any promise in humans. The non-competitive glutamate receptor antagonists Amantadine and Memantine (acting on the NMDAR) did not aid addicts to abstain from cocaine (Kampman et al., 2006; Collins et al., 2007). Although promising in rodent cocaine studies and successful for human alcohol dependence, the drug Acamprosate (NMDA type Glu receptor antagonist and GABAa receptor agonist), proved ineffective for human cocaine addicts (Kampman et al., 2011). Some success was had with *N*-acetylcysteine, which promotes the replacement of intracellular glutamate for cysteine via anti-porters and so reduced glutamate transmission. In a pilot study, it was found to diminish the taking of cocaine by the majority of patients after treatment in the study (Mardikian et al., 2007). This may encourage future studies aiming to tone down Glu transmission in cocaine addiction.

Furthermore, in the early 1990s, a cocaine vaccine was developed. It was believed that the production of antibodies against the cocaine molecules could block its effects and thus help maintain abstinence. Unfortunately, getting patients to produce efficient levels of antibodies proved to be a great limit to this approach. A vaccine is currently under phase II multi-site clinical trials, but some reservations have been voiced regarding ethics of vaccines against addiction being used as an prevention or as a treatment (Young et al., 2012).

In summary, a considerable number of human and pre-clinical animal studies focus on restoring perturbations in the balance of DA and Glu transmission as a therapy for cocaine addiction, but from animal studies we now know that ERK signaling is a key coordinator in this system. We propose that D1R/GluN1 oligomers could participate also in this synergy. While blocking ERK activation by drugs is validated in animal models, its potential for human treatment is limited as it is a kinase implicated in a wide range of cellular processes. Instead, we content that targeting ERK signaling and its downstream partners (MSK-1 and histone phosphorylation, Elk-1 phosporylation) within D1R-MSN specifically is a promising strategy. This strategy could include the signaling events that are at the crossroad of D1R and NMDAR synergism in response to cocaine (see **Figure 1**).

## Conflict of Interest Statement

The authors declare that the research was conducted in the absence of any commercial or financial relationships that could be construed as a potential conflict of interest.
